# Modeling event‐related heart period responses

**DOI:** 10.1111/psyp.12622

**Published:** 2016-02-05

**Authors:** Philipp C. Paulus, Giuseppe Castegnetti, Dominik R. Bach

**Affiliations:** ^1^ Department of PsychiatryPsychotherapy, and Psychosomatics, University of ZurichZurichSwitzerland; ^2^ Neuroscience Center Zurich, University of ZurichZurichSwitzerland; ^3^ Department of PsychologyTechnische Universität DresdenDresdenGermany; ^4^ Wellcome Trust Centre for Neuroimaging, University College LondonLondonUK

**Keywords:** Psychophysiological model, Statistical analysis, Heart period, Cardiovascular, Emotion, Young adults

## Abstract

Cardiac rhythm is generated locally in the sinoatrial node, but modulated by central neural input. This may provide a possibility to infer central processes from observed phasic heart period responses (HPR). Currently, operational methods are used for HPR analysis. These methods embody implicit assumptions on how central states influence heart period. Here, we build an explicit psychophysiological model (PsPM) for event‐related HPR. This phenomenological PsPM is based on three experiments involving white noise sounds, an auditory oddball task, and emotional picture viewing. The model is optimized with respect to predictive validity—the ability to separate experimental conditions from each other. To validate the PsPM, an independent sample of participants is presented with auditory stimuli of varying intensity and emotional pictures of negative and positive valence, at short intertrial intervals. Our model discriminates these experimental conditions from each other better than operational approaches. We conclude that our PsPM is more sensitive to distinguish experimental manipulations based on heart period data than operational methods, and furnishes a principled approach to analysis of HPR.

Cardiac rhythm generators form a semiautonomous system, which is nevertheless modulated by sympathetic and parasympathetic afferents (Berntson, Quigley, & Lozano, 2007). Consequently, observing heart rhythm may allow inference on states of the central nervous system. On a time scale of minutes, such inference is engendered in algorithms to quantify tonic parasympathetic state from heart rate variability (Allen, Chambers, & Towers, 2007; Berntson, Cacioppo, & Quigley, 1993; Berntson et al., 2007). On the other hand, phasic autonomic input can be inferred from heart period responses (HPR) over seconds (Bradley, Codispoti, Cuthbert, & Lang, 2001; Hodes, Cook, & Lang, 1985). Operational analysis methods for such phasic responses compare peaks and troughs of an interpolated heart period time series with a prestimulus baseline (e.g., Hodes et al., 1985). Studies using such methods have suggested various HPR patterns, depending on the type of stimulus. Arousing pictures elicit a triphasic HPR, comprising two decelerations and an acceleration in alternating order (Bradley et al., 2001; Hodes et al., 1985). Amplitudes of primary deceleration and subsequent acceleration appear influenced by stimulus valence when pictures are presented for 6 s (Bradley et al., 2001), but not for short presentations (i.e., < 1 s; Codispoti, Bradley, & Lang, 2001; Ruiz‐Padial, Vila, & Thayer, 2011). An inverted response pattern was identified for aversive auditory stimuli with two accelerations and a deceleration in alternating order (Reyes del Paso, Godoy, & Vila, 1993). In an auditory oddball task, participants showed cardiac deceleration while listening to standard tones. The presentation of an oddball tone elicited cardiac acceleration (Weber, Van der Molen, & Molenaar, 1994).

To relate such responses to central neural input, pharmacological studies suggest particular time courses of sympathetic and parasympathetic influence. Specifically, primary acceleration and deceleration are not affected by the drug metoprolol, which blocks sympathetic input (Reyes del Paso et al., 1993). On the other hand, later response components appear to result from an interplay of both branches of the autonomic nervous system, but still with a major parasympathetic influence (Berntson et al., 2007; Reyes del Paso et al., 1993).

Currently used operational peak‐scoring approaches embody implicit causal models of how precisely central states cause cardiac responses, as apparent, for example, in the choice of time windows to be analyzed (Bach & Friston, 2013). However, there is no consensus on these implicit models—partly because they are never explicitly tested— and their application often appears to be driven by experiment‐specific considerations. Here, we take a more principled approach to overcome these limitations. We seek to create an explicit psychophysiological model (PsPM) that describes, in mathematical form, how autonomic input generates cardiac responses. This model can then be inverted to retrieve experiment‐specific estimates of this autonomic input. This parallels a previously developed, model‐based method for analyzing skin conductance responses, which has been shown to outperform operational approaches in recovering a known central state (Bach, 2014; Bach, Daunizeau, Friston, & Dolan, 2010; Bach, Flandin, Friston, & Dolan, 2009; Bach & Friston, 2013; Bach et al., 2013) and has been used in various experimental contexts (Fan et al., 2012; Hayes et al., 2013; Nicolle, Fleming, Bach, Driver, & Dolan, 2011; Sulzer et al., 2013; Talmi, Dayan, Kiebel, Frith, & Dolan, 2009).

Given relatively sparse knowledge on the precise biophysics of heart rate modulation, our PsPM remains phenomenological as in our previous approach to skin conductance responses (Bach, Friston, & Dolan, 2010). Hence, it departs from other cardiac modeling work that provided more biophysical realism (Riedl et al., 2008; Somsen, Molenaar, Molen, & Jennings, 1991; Żebrowski et al., 2007) but did not as yet allow inference on autonomic input, possibly due to the complexity of these models apparent in the number of free parameters.

To quantify phasic changes in cardiac chronotropy, two measures are common: heart rate (beats per minute) or heart period (milliseconds). Heart period appears to relate to autonomic input linearly, as revealed in experiments in which autonomic nerves in rodents were electrically stimulated with varying frequencies to elicit heart period changes (Berntson, Cacioppo, & Quigley, 1995). Since our PsPM is developed to quantify autonomic input, it is parsimonious to base this inference on heart period. As in previous work, we interpolate discrete heart period values to create continuous time series (cf. Allen et al., 2007; Hodes et al., 1985; Koers, Mulder, & van der Veen, 1999). We note that this strategy produces time series almost identical to those derived with alternative approaches proposed in the literature (Graham, 1978; Koers et al., 1999).

Crucially, the aim of our model is not to explain all variance in the heart period time series but to estimate autonomic input from the data. We evaluate this by assessing the model's predictive validity. Because ground truth (i.e., the true autonomic input into the heart) cannot be concurrently measured, we take the approach to experimentally create categorically different situations, which we then seek to correctly identify from the model's parameter estimates (Bach & Friston, 2013). To develop the model, we elicit different sympathetic and parasympathetic activation patterns in three experimental tasks, which are based on previous operational research. We then evaluate the model's predictive validity in an independent experiment.

In summary, we hypothesized that sensory and attentional processing of auditory and visual material elicits a general increase in parasympathetic input leading to cardiac deceleration, followed by an increase in sympathetic input to the heart, which is responsible for an acceleration. For the aversive white noise sounds, we expect to replicate the patterns found by Reyes del Paso et al. (1993) that comprised two accelerations and decelerations in alternating order. Hence, we expect categorically different response patterns both on a descriptive and on a statistical level.

## Method

### Design and Participants

For Experiment 1 and 2, we recruited 61 healthy unmedicated participants from the general population (32 female, 29 male, mean age ± *SD*: 26 ± 4.6 years, range 18–36 years). Average temperature of the testing room was 25.7°C (range 24.5–26.7°C), and average humidity was 43% (range 27%–66%). Experiment 1 elicited HPR by repeated presentation of aversive auditory stimuli. We excluded four participants due to technical failures and one because muscle artifacts made QRS detection impossible. The same 61 participants then underwent an auditory oddball task. Two participants did not complete this Experiment 2, and a single different participant was excluded because muscle artifacts made QRS detection impossible. Average temperature of the testing room was 25.7°C (range 24.6–26.8°C), and average humidity was 42.6% (range 27%–67%). The order of the two experiments was fixed for all participants. Experiment 3 followed a one‐way factorial design with three levels (neutral pictures, positive pictures, negative pictures), and we included an independent sample of 23 healthy unmedicated participants (13 female, 10 male, mean age ± *SD*: 26 ± 4.6 years, range 18–36 years) from the general population. Average temperature of the testing room was 24.8°C (range 23.3–26.8°C), and average humidity was 32.6% (range 25%–38%). Finally, Experiment 4 utilized a one‐way factorial design with four levels (negative pictures, positive pictures, intense auditory simulation, mild auditory stimulation). Nineteen healthy unmedicated participants were recruited from the general population (11 female, 8 male, mean age ± *SD*: 26 ± 5.4 years, range 18–38 years). One participant was excluded due to the incidental finding of a possible cardiac condition. Average temperature of the testing room was 25.0°C (range 23.5–26.2°C), and average humidity was 37.5% (range 35%–41%). All participants gave written informed consent and received monetary compensation for their participation. All experimental protocols, including the form of taking consent, were approved by ethics committees (Experiments 1, 2: Ethikkommission der Charité Universitätsmedizin, Berlin, Germany; Experiments 3, 4: Kantonale Ethikkommission, Zurich, Switzerland).

### Stimuli and Procedure

#### Experiment 1

Twenty broadband white noise sounds of 1‐s duration (10‐ms onset and offset ramp, ∼85 dB sound pressure level) were delivered via headphones (PX‐660 Pro Luxe, Fujikon, Hong Kong, China). The intertrial interval (ITI) was randomly chosen to be 29 s, 34 s, or 39 s. A fixation cross was visible on the screen all the time, and participants were instructed to press a key on a standard computer keyboard as soon as they heard the sound. All stimuli were presented in one block.

#### Experiment 2

After finishing Experiment 1, the same participants heard, every second, one of two sine tones (50‐ms length, 10‐ms onset and offset ramp, ∼75 dB, 440 Hz or 460 Hz) via headphones. The participants were instructed to press a key on a standard computer keyboard on hearing one of the 10 oddball tones, the pitch of which was balanced across participants. A fixation cross was visible on the screen all the time. The number of standard tones was arranged to realize a random ITI between the oddball tones of 29 s, 34 s, or 39 s. All stimuli were presented in one block.

#### Experiment 3

Participants were presented with the 16 least arousing neutral, 16 most arousing negative, and 16 most arousing positive (excluding explicit nude) pictures from the International Affective Picture System (IAPS; Lang, Bradley, & Cuthbert, 2005). The selected pictures were the same as in previous studies (Bach, 2014; Bach et al., 2013; Bach, Seifritz, & Dolan, 2015). Participants were instructed to press the cursor up or down key on a standard computer keyboard to indicate whether they liked the stimulus or not. Stimuli were presented for 1 s with a random ITI of 43 s, 45 s, or 47 s. All pictures were presented in one block, and a fixation cross was visible between two consecutive pictures.

#### Experiment 4

A total of 120 white noise sounds with a sound pressure level of ∼85 dB or ∼65 dB (1‐s length, 10‐ms onset and offset ramp) were delivered via headphones, and the 60 most arousing negative and 60 most arousing positive (excluding explicit nude) pictures from the IAPS were presented for 1 s, all in randomized order. Before and after picture presentation, as well as during the white noise trials, a fixation cross was visible on the computer screen. Participants were instructed to press the cursor up or down key on a standard computer keyboard to indicate whether they liked the sound or picture. The stimuli were presented with a variable ITI of 10 s ± 6 s in three blocks with a pause of 90 s between two consecutive blocks.

#### Common settings

All experiments were programmed in Cogent (Version 2000 v1.25; www.vislab.ucl.ac.uk/Cogent) in MATLAB (MathWorks, Natick, MA). Experiments took place in dimly lit and sound‐insulated testing rooms. Participants were placed in a comfortable chair with armrests and were asked not to move during the experimental sessions to minimize movement artifacts. Remaining movement and muscle artifacts were manually corrected by visual inspection of all IBIs larger or shorter than the mean IBI ± 2 *SD*.

### Apparatus

Electrocardiogram (ECG) was recorded using four electrodes attached to the limbs. The lead (I, II, III) or augmented lead (aVR, aVL, aVF) configuration with the highest R spike was visually identified by the experimenter and recorded. Data were preamplified and 50 Hz notch‐filtered using a Coulbourn isolated five‐lead amplifier (Model V75‐11, Coulbourn Instruments, Allentown, PA), digitized at 1000 Hz using a Dataq card (Model DI‐149 A/D, Dataq Inc., Akron, OH), and recorded with Windaq (Dataq Inc.).

### Data Preprocessing

Preprocessing was carried out in MATLAB 8.3 (MathWorks). ECG data was first filtered with an antialias Butterworth low‐pass filter (second‐order, cutoff 100 Hz) and down sampled to 200 Hz. A modified offline implementation of the Pan and Tompkins (1985) real‐time QRS detection algorithm was then used to identify QRS complexes (see Appendix for changes from the original algorithm and detection accuracy). A visual correction of all interbeat intervals (IBIs) longer or shorter than the average IBI ± 2 *SD* per dataset was performed to further increase detection accuracy. The correction was performed on the continuous ECG dataset, and the rater was blind to event timing and types. Each IBI was assigned to its following heartbeat. The time series was then linearly interpolated to achieve a sampling rate of 10 Hz. To remove slow drifts, smooth the angles introduced by the interpolation, and reduce the influence of potentially remaining misdetections, the time series was filtered with a second‐order Butterworth band‐pass filter with cutoff frequencies of .01 and 2 Hz, respectively. The QRS detection algorithm (scr_ecg2hb), the interpolation function (scr_hb2hp), the graphic user interface for visual inspection of the data (scr_display), and the tool to manually correct falsely detected QRS complexes (scr_ecg2hb_qc) are included in the MATLAB toolbox Psychophysiological Modelling (PsPM), which can be obtained under the GNU General Public License from http://pspm.sourceforge.net

### Peak Scoring

Operational analysis was conducted according to the protocol of Hodes et al. (1985). We selected this approach because it uses time windows that well resemble those of primary deceleration, acceleration, and secondary deceleration to briefly presented stimuli (cf. Codispoti et al., 2001). We computed the baseline value as mean over a 1‐s baseline interval (B, −1 – 0 s) and performed peak scoring in the respective time windows to obtain primary deceleration (D1, 0 – 2 s), acceleration (A, 2 – 5 s), and secondary deceleration (D2, 5 – 8 s). Time windows are specified in relation to the occurrence of the stimuli. We then computed the values for the primary deceleration (B‐D1), acceleration (A‐B), secondary deceleration (B‐D2), acceleration relative to primary deceleration (A‐D1), and secondary deceleration in relation to acceleration (A‐D2). Furthermore, we computed peak deceleration and peak acceleration from baseline over the complete trial duration.

### Model Development and Statistical Analysis

To keep the model simple, we treated the heart period time series as output of a set of linear time invariant (LTI) systems. This type of system is an approximation to biophysical reality with two main features: (1) the response of the system to the same input is always the same (i.e., the output depends on the input only), and (2) the response to two inputs is the sum of the responses to the individual inputs. An LTI system is unambiguously specified by its response function (RF). Inputs into these LTI systems are specified by a neural model. Here, we assume that short stimuli elicit very brief neural inputs into the systems. This provides for a simple inversion scheme. By convolving the RF with a vector of impulse functions at the onsets of each event type, we obtain predicted time series, which are then combined into one design matrix to specify a general linear model (GLM). Inverting this GLM yields estimates for the amplitude of HPR components (Bach et al., 2009; Friston, Jezzard, & Turner, 1994), each of which can be interpreted as amplitude of an autonomic input component. While this is one of the simplest approaches to PsPM, we note that assumption 2 of the LTI properties of the cardiovascular system may be unrealistic since the range of physiologically possible heart periods is limited, and the system will therefore quickly saturate. This is why, in contrast to models for skin conductance responses (Bach et al., 2009), we do not aim at estimating overlapping responses. Hence, a possible violation of the linearity assumption is relatively unproblematic for the present work.

In our phenomenological approach, we sought to determine a basis set of RFs from experimental data, to define a set of LTI systems. This was conducted in a sequential procedure. The data were epoched from 2 s before stimulus onset to 29 s after stimulus onset and mean centered, to compute a principal component analysis (PCA) over all epochs from all participants of Experiments 1–3. By inspection of the scree plot, the first three principal components (PCs) were identified as relevant and extracted. We then modeled shapes and latencies of all individual peaks within these three PCs by fitting Gaussian functions to the peaks. Because of the interpolation, the cardiac response can appear to start before stimulus onset. To account for this, the RFs also included a prestimulus interval of 5 s.

To test whether a modeled peak qualified as a RF, we subjected it to a testing procedure in which we started with a single RF and included further RFs. Specifically, we created a first‐level GLM for each participant by convolving the event onsets with the specific subset of RFs, and extracted estimates of the response amplitudes for each RF and condition from the continuous heart period data. In order to qualify as RF, parameter estimates for this RF were either required to depict a stable response (i.e., acceleration or deceleration) across all experiments, tested by a one‐sample *t* test, or to add to the predictive validity of the model. This second criterion was evaluated by subjecting the parameter estimates to a between‐subjects analysis of variance (ANOVA) testing for a main effect of experimental condition. Hence, the following steps were taken:

##### Step 1

Test all potential RFs modeled from PCs individually and retain each single RF if it depicts a stable acceleration or deceleration across all experiments or if it allows separation of at least two of the three experiments. All potential RFs fulfilled one of these criteria.

##### Step 2

Take the chronologically first RF from Step 1 and combine it with the chronologically second RF from Step 1, or with any other RF that overlaps with this second RF. Orthogonalize each set in temporal order, using a Gram‐Schmidt algorithm. Retain the best set if additional RFs allow the separation of the three experiments.

##### Step 3–5

Take the current set, and add the chronologically next untested RF from Step 1, or any other RF that overlaps in time with this second RF. Repeat the strategy of Step 2, and retain the best set if additional RFs allow the separation of the three experiments.

Trials from Experiments 1 and 2 that came from the same participants were treated as independent to reduce complexity of exploratory tests. All analyses were performed using PsPM and in‐built MATLAB functions.

### Model Evaluation

For model evaluation, we set up one GLM per participant and extracted parameter estimates for each condition and RF. Further analysis was carried out in SPSS 21.0 (IBM, Armonk, NY). In order to compare our novel method with a standard peak scoring approach, we computed Akaike's information criterion (AIC) as an approximation to model evidence with the MATLAB function scr_predval that is part of the PsPM toolbox. Note that our approach here is to predict experimental conditions from the parameter estimates of the model, or from peak amplitudes obtained by peak scoring. Hence, the dependent variable in this context are the experimental conditions and our independent variable are estimates of HPR amplitude obtained by the respective methods. This procedure is similar to approaches used to evaluate predictive validity in the context of other model‐based methods (e.g., Bach, 2014). AIC is the negative log likelihood of the model, plus a complexity term that was the same for all tested predictive models (Burnham, 2004). An absolute AIC difference of  > 3 is often regarded as decisive, by analogy to a classic *p* value. If a classic test statistic falls into the rejection region, the probability of the data given the null hypothesis is *p* < .05. Unlike *p* values, AIC scores allow quantification of evidence in favor of a null hypothesis. For an AIC difference  > 3, the probability of the null hypothesis given the data is 1/exp(3) ≲ .05 (Penny, Stephan, Mechelli, & Friston, 2004; Raftery, 1995). We also computed *t* values for illustration of our results.

## Results

### Manipulation Check

**Figure 1:**
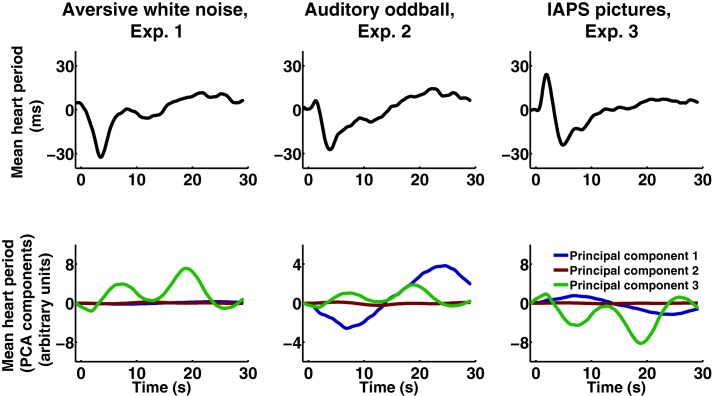
Heart period responses. Upper: Mean phasic response over all participants of each experiment. Lower: Results of PCA over all participants of Experiments 1–3. Principal components are weighed by their mean factor loading per experiment.

On the basis of operational measures obtained with the protocol by Hodes et al. (1985), we found a statistically significant main effect of experimental condition for secondary deceleration (B‐D2; *F*(2,134) = 7.735, *p* = .001) and secondary deceleration in relation to acceleration (A‐D2; *F*(2,134) = 7.104, *p* = .001). In a different analysis scheme, the same effect was observed in the peak deceleration from baseline, *F*(2,134) = 5.302, *p* = .015. Table 1 contains a summary of average parameters for the individual experiments and shows that accelerations and decelerations were present across all experiments. In conjunction with descriptive statistics (see Figure 1, upper panels), the results suggest that we were able to provoke categorically different responses in each of the three experiments. To rule out potential biasing effects of serial dependency due to the fixed order of Experiment 1 and 2, we compared average heart periods from the first trials of both experiments and found no evidence for serial dependency, *t*(112) = −0.317, *p* = .752.

**Table 1:** Mean Accelerations and Decelerations in the Three Experiments

	Experiment 1		Experiment 2		Experiment 3	
Parameter	*M* (ms)	*p*	*M* (ms)	*p*	*M* (ms)	*p*
Peak deceleration	86.1	< .001	87.9	< .001	115.1	< .001
Peak acceleration	−112.9	< .001	−103.3	< .001	−110.7	< .001
B‐D1	−15.0	< .001	−14.7	< .001	−18.2	< .001
A‐B	−0.4	.739	−1.5	.474	−0.3	.858
B‐D2	7.8	.035	9.2	.009	31.3	< .001
A‐D1	15.4	< .001	16.1	< .001	18.4	< .001
A‐D2	8.3	.022	−10.7	.051	−31.5	< .001

### Model Development

The first three PCs of 2,804 phasic responses from 84 participants and three experiments explained 37.4% of total variance. Exploratory ANOVAs on the PC loadings yielded a statistically significant main effect of experimental condition for PC 3, *F*(2,134) = 8.63, *p* < .001. There were no statistically significant main effects of experimental condition for PC 1, *F*(2,134) = 2.21, *p* = .114, and PC 2, *F*(2,134) = 0.01, *p* = .986. Descriptive and PCA results are depicted in Figure 1.

Physiological considerations suggest a relative dominance of parasympathetic influences in the dataset that might be coupled over all three experiments and therefore load on the same PC, although in theory be physiologically independent. To identify individual physiological response functions rather than their combination, we modeled and tested all individual peaks within the PCs as potential RFs. The complete procedure is summarized in the Appendix. In short, six RFs were retained in the final model, each of which allowed differentiation of at least two of the three experiments. The resulting model is depicted in Table 2.

**Table 2:** Results of the T Tests and ANOVA on the Resulting Final Model from Model Development

	*t* test			ANOVA		
RF	*t*(136)	*p*	Direction of response	*F*(2,134)	*p*	Post‐hoc contrasts
1	2.81	.006	+	5.43	.005	(E1 = E2) < E3
2	−7.88	< .001	−	14.18	< .001	(E1 = E3) < E2
3	−3.56	.001	−	30.47	< .001	E1 > (E2 = E3)
4	−7.73	< .001	−	6.87	.001	E1 < E2
5	−6.92	< .001	−	6.75	.002	(E1 = E3) > E2
6	1.19	.237	*n.s*.	7.66	.001	E1 > (E2 = E3)

*Note*. Experiment 1: auditory white noise experiment; Experiment 2: auditory oddball experiment; Experiment 3: IAPS pictures. RF = response function; *t* test = test for the general direction of the response across experiments; direction of response = direction of the general response (minus signs indicate accelerations, plus signs indicate decelerations); ANOVA = analysis of variance testing for a main effect of experimental condition.

**Figure 2:**
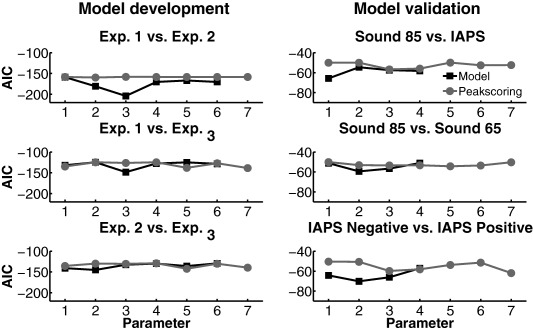
Predictive validity expressed as AIC for model‐based analysis and operational analysis. Smaller AIC values indicate higher predictive validity of the respective parameter. Operational parameters are peak deceleration from baseline (Parameter 1), peak acceleration from baseline (Parameter 2), primary deceleration (B‐D1, Parameter 3), acceleration (A‐B, Parameter 4), secondary deceleration (B‐D2, Parameter 5), acceleration in relation to primary deceleration (A‐D1, Parameter 6), and secondary deceleration in relation to acceleration (A‐D2, Parameter 7). Model‐based parameters are parameter estimates for RFs 1 to 6. Left: AIC for linear contrasts of model development. Right: AIC for linear contrasts of model validation.

To determine the extent to which the operational method and the model were able to recover the hidden states from the data, we computed AIC for all three linear contrasts between the experiments. AIC scores of relative model evidence for operational and model‐based analyses are depicted in the left panels of Figure 2.

### Model Validation

Finally, we tested the predictive validity of the model in an independent experiment with shorter ITI. We presented white noise sounds with two intensities (65 dB and 85 dB), and arousing IAPS pictures (negative and positive). To avoid estimation of overlapping responses, we discarded RF5 and 6 from the model. A first linear contrast was planned to replicate the comparison between Experiment 1 and Experiment 3. From model development, we expected to observe statistically significant larger parameter estimates for IAPS pictures as compared to loud sounds for RF1, and statistically significant larger estimates for loud sounds as compared to IAPS pictures for RF3. A second linear contrast tested the intensity effect in the auditory white noise conditions (85 dB vs. 65 dB). We had no directional hypothesis for Contrast 2, but predicted from operational literature a difference of the two conditions due to the averseness of 85 dB sounds as compared to 65 dB sounds. Finally, a third contrast tested the valence effect in the IAPS pictures (negative vs. positive). In Experiment 3, we had observed a trend for RF1 in the contrast comparing negative and positive IAPS pictures, *t*(22) = 1.952, *p* = .064, and expected that, due to the increased number of trials in the validation experiment, we would retain statistically significant larger parameter estimates for negative as compared to positive pictures in RF1. The results of the planned contrasts are depicted in Table 3. Our hypothesis for Contrast 1 was confirmed for RF1: IAPS pictures elicited statistically significant larger early responses in RF1 than loud sounds, while the test for RF3 only showed a trend toward significance. Results for Contrast 2 showed a statistically significant averseness effect for RF2 and a trend toward statistical significance for RF3. The results for Contrast 3 show statistically significant larger parameter estimates for RF1 for negative pictures than for positive pictures, as hypothesized. Furthermore, in this contrast, we observed statistically significant differences for RF2 and RF3, as well as a trend toward statistical significance for RF4. On the other hand, operational indices yielded a statistically significant difference only for the comparison of negative and positive IAPS pictures in the primary deceleration in relation to baseline, and the secondary deceleration in relation to the acceleration. A trend toward statistical significance was also apparent for acceleration in relation to baseline. Parameter estimates from our model yielded statistically significant differences for all three contrasts in at least one RF, and operational parameters only yielded statistically significant differences for Contrast 3. To directly compare operational and model‐based analysis, we computed estimates of predictive validity for the planned contrasts. The results are depicted in Figure 2 (right panels) and show similar or lower AIC (i.e., higher predictive validity) for model‐based analysis as compared to operational analysis in all three comparisons and for all tested parameters.

**Table 3:** Results of the Linear Contrasts for the Validation Experiment

Planned contrast	Parameter	Operational analysis		Model‐based analysis		Mean parameter estimates	
		*t*(17)	*p*	*t*(17)	*p*	${\bar{x}}_{1}$	${\bar{x}}_{2}$
Sound 85 vs. IAPS	1	0.13	.901	−3.06	.007*	7.99	68.28
	2	0.18	.857	−1.54	.143	−59.66	−40.85
	3	−1.88	.078	1.99	.063	74.47	7.67
	4	1.75	.098	−2.10	.051	23.12	155.77
	5	−0.03	.979	–	–	–	–
	6	−1.15	.267	–	–	–	–
	7	1.10	.287	–	–	–	–
Sound 85 vs. Sound 65	1	0.34	.738	0.80	.438	7.99	−7.05
	2	1.29	.215	2.27	.036*	−59.66	−96.54
	3	−1.34	.199	1.88	.077	74.47	24.26
	4	1.31	.207	0.77	.453	23.12	−14.97
	5	1.48	.156	–	–	–	–
	6	−1.36	.193	–	–	–	–
	7	−0.43	.672	–	–	–	–
IAPS negative vs. IAPS positive	1	0.49	.631	2.88	.010*	83.07	53.49
	2	0.62	.543	3.59	.002*	−17.32	−64.38
	3	−2.32	.033*	−3.12	.006*	−18.18	33.53
	4	2.09	.052	1.95	.068	203.18	108.37
	5	−1.39	.182	–	–	–	–
	6	−0.87	.395	–	–	–	–
	7	2.60	.019*	–	–	–	–

*Note*. Operational analysis: Parameter 1 = peak deceleration; Parameter 2 = peak acceleration; Parameter 3 = B‐D1; Parameter 4 = A‐B; Parameter 5 = B‐D2; Parameter 6 = A‐D1; Parameter 7 = A‐D2. Model‐based analysis: Parameter 1–5 = parameter estimates for RF 1–5.

*p < .05.

## Discussion

In this article, we present a novel method to analyze event‐related HPR. This method is built on a phenomenological PsPM of how autonomic input influences heart period. Three experiments with long ITIs are analyzed to develop the model, and show that both operational approaches and the new model can recover experimental conditions from the data. This result is confirmed in an independent validation experiment with fewer participants and shorter ITIs. Here, parameter estimates of the novel model‐based approach successfully separate experimental conditions in all three planned contrasts. In contrast, indices from classic analysis show statistically significant differences only in one out of three linear contrasts. Indeed, for all three contrasts and all parameters/indices, predictive validity is higher for the model‐based method. In conjunction, these results indicate that our model is more sensitive than a standard method for analyzing event‐related HPR.

The final model contains six RFs, for each of which an amplitude parameter is estimated. Descriptively, each of these parameters quantifies the magnitude of the respective response component. However, we can interpret their values as amplitude of an autonomic input component, in line with previous operational approaches. How these autonomic components map onto the known physiology of the sympathetic and parasympathetic nervous system cannot yet be unambiguously answered from the present work. One may tentatively link the first two RFs to parasympathetic input (Reyes del Paso et al., 1993), while later response components may represent input from either or both of the two branches of the autonomic nervous system, in line with operational indices at similar poststimulus times. In order to investigate the causes of these responses more closely, one would ideally use pharmacological manipulations. It might then be possible to distinctly assign the respective RFs to underlying parasympathetic or sympathetic input. Also, the first RF appears to start before stimulus onset. While this may be a result of the interpolation procedure, it could also reflect anticipatory processes. Experiments with more varied ITIs may be able to resolve this question.

The model in its present form is a first attempt to apply the framework of psychophysiological modeling (Bach & Friston, 2013) to event‐related HPR. It emerges that some aspects need to be reviewed more closely in the future. First, the model was developed to infer central states from observed data. It has been demonstrated that methods optimized to accurately describe observed data often do not achieve this inference (Bach, 2014; Bach et al., 2013). However, our approach leaves a major proportion of variance in the observed signal unexplained. In particular, the heart period time series may be dominated by respiratory sinus arrhythmia (RSA, Berntson et al., 1993). With many repetitions of an event as in the present experiments, one is able to effectively average out such variance. However, an explicit model of RSA could make the method more powerful in analyzing data from experiments with few trials. Hence, PsPMs of the interaction between respiration and heart period may be advantageous.

Further, the model has been developed on stimuli of a fixed duration of 1 s. As the shape of the cardiac response depends strongly on the duration of the input (cf. Bradley et al., 2001; Codispoti et al., 2001), our method in its current form is only applicable to experiments with brief stimulus presentation durations. Future work will address whether the model also generalizes to experiments with longer stimulus durations. Also, the two central assumptions, linearity and time invariance, will have to be firmly tested. As demonstrated for other methods, the linearity assumption strongly depends on the duration of the ITI (Bach, Flandin, Friston, & Dolan, 2010). Indeed, in the case of responses very close in time, the system might saturate, thus making the linearity assumption unrealistic.

In the present implementation of the model, parameters are directly estimated from the interpolated IBI time series that might be corrupted by faulty detected QRS complexes. While the automatic version of our offline implementation of the QRS detection algorithm achieved 99.66% detection accuracy, the proportion of faulty detected QRS complexes was as high as 2% for one dataset. This is why we used a graphic user interface to allow visual correction of all IBIs that fall outside the average IBI ± 2 *SD*. Furthermore, we introduced liberal filtering of the data at cutoff frequencies of .01 and 2 Hz. To further enhance data quality and eliminate the necessity for visual inspection, future work should also aim at the optimization of the filter settings. For skin conductance data, optimizing filter parameters has been shown to increase predictive validity of model‐based analysis (Bach et al., 2013; Staib, Castegnetti, & Bach, 2015). An additional technical limitation imposed by the current implementation of the QRS detection algorithm is the temporal resolution of 5 ms.

A further important aspect is model complexity. In its current form, the model comprises six RFs. We deliberately separated response components to allow their independent investigation. However, it might well be the case that some of them are in fact physiologically inseparable and relate to the same autonomic input component. To reduce the number of RFs, future work should test for inseparable coupling between RFs. Physiologically coupled responses could then be combined into one regressor to reduce model complexity.

As a practical recommendation, before such issues are resolved, we would like to suggest two points for analyzing experimental data. First, because the linearity assumption of the model is brittle, one may prefer using only RFs that peak within the minimum ITI of an experiment. Secondly, to avoid correcting for multiple comparison when using more than one RF, it may be advisable to form clear hypotheses in which of the six RFs to expect an effect of an experimental manipulation, for example, based on operational literature. If clear hypotheses can be formulated, it may also be possible to only include the respective RF into the model.

To summarize, we present a PsPM for event‐related heart period responses, based on a set of linear time invariant systems. Inversion of this model yields parameter estimates that better separate known psychological states than an operational approach. With this work, we hope to have inspired renewed interest in the use of heart beat data to infer central, neural, or psychological states.

## Figures and Tables

**Figure 3:**
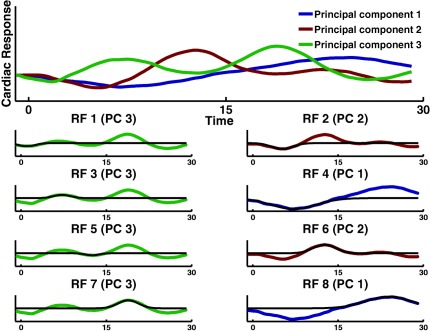
Principal components and fitted Gaussian functions. Top: First three principal components. Bottom: RFs 1–8 plotted with the principal components they were modeled from. Fits are depicted in black, principal components in their respective color.

## Appendix 1

### 1 Pan and Tompkins (1985) QRS Detection Algorithm

Pan and Tompkins (1985) published an online algorithm to identify QRS complexes in ECG signals. The algorithm analyzes slope, amplitude, and width of QRS complexes. It uses a digital band‐pass filter to reduce noise. Thresholds are periodically adjusted to low values. For an offline implementation, we made some adjustments to this algorithm. In the original algorithm, Pan and Tompkins used cascaded integer filters to realize an approximation to the pass band from 5 to 15 Hz. The cascaded integer filters were replaced by a second‐order Butterworth filter realizing cutoff frequencies of 5 and 15 Hz. Instead of real‐time derivation, numerical differentiation as implemented in MATLAB was used. To increase the accuracy of detection, minimal and maximal heart rate values were set to be 20 bpm (IBI of 3,000 ms) and 200 bpm (IBI of 300 ms), respectively. The marker identifying the QRS complex is positioned at the rising edge of the integrated waveform signal that corresponds with the position of the R spike of the ECG.

Approximately 20% (24) of datasets of Experiments 1–3, containing 24,398 heartbeats, were randomly selected and visually inspected. The algorithm yielded 82 detection errors of which 45 were false positive and 37 were false negative beats. This leads to a sensitivity of 99.85%, a total accuracy of 99.66%, and a positive predictive value of 99.82%.

### 2 Model Development

The potential RFs were modeled from the first three principal components obtained by PCA (cf. Figure 3). In Step 1, parameter estimates for each RF were estimated individually. This was done to test whether the RFs were generally carrying predictive information or depicted a stable acceleration or deceleration across the three experiments. Since at least one of the two criteria was fulfilled for all response functions, we tested all RFs in the following steps. In each of these steps, RFs with similar timing—suggesting that they depict similar physiologic response components—were included into the model and tested against each other. Only those RFs that qualified in the individual steps (i.e., had a larger *F* value in the ANOVA testing for a main effect of experimental condition) were retained. In order to achieve uncorrelated regressors, the models in Steps 2–5 were orthogonalized in temporal order of the modeled peaks. Results of model development are depicted in Table 4.

**Table 4:** Results of Model Development

		*t*‐test			ANOVA		
Step	RF	*t(*136)	*p*	Direction of response	*F*(2,134)	*p*	Post‐hoc contrasts
1	1	3.23	.002	+	9.97	< .001	(E1 = E2) < E3
	2	−6.01	< .001	–	25.72	< .001	(E1 = E3) < E2
	3	−5.10	< .001	–	15.37	< .001	E3 < E1 < E2
	4	−7.41	< .001	–	29.13	< .001	(E1 = E3) < E2
	5	−5.60	< .001	–	4.36	.015	E1 < E2
	6	−5.77	< .001	–	6.84	.001	E1 < E2
	7	−5.56	< .001	–	6.51	.002	E1 < E2
	8	4.98	< .001	+	33.03	< .001	E1 > (E2 = E3)
2	1	3.07	.003	+	8.11	< .001	(E1 = E2) < E3
	2	−7.78	< .001	–	13.80	< .001	(E1 = E3) < E2
	1	3.04	.003	+	7.76	.001	(E1 = E2) < E3
	3	−6.78	< .001	–	5.11	.007	*E2 > E3*
	**1**	**3.03**	**.003**	**+**	**7.58**	**.001**	**(E1 = E2) < E3**
	**2**	**−7.80**	**< .001**	**–**	**13.88**	**< .001**	**(E1 = E3) < E2**
	**3**	**−3.48**	**.001**	**–**	**30.90**	**< .001**	**E1 > E3 > E2**
3	**1**	**2.88**	**.005**	**+**	**6.18**	**.003**	**(E1 = E2) < E3**
	**2**	**−7.85**	**< .001**	**–**	**14.05**	**< .001**	**(E1 = E3) < E2**
	**3**	**−3.53**	**.001**	**–**	**30.81**	**< .001**	**E1 > (E2 = E3)**
	**4**	**−7.71**	**< .001**	**–**	**7.16**	**.001**	**E1 < E2**
4	1	2.87	.005	+	6.11	.003	(E1 = E2) < E3
	2	−7.86	< .001	–	14.05	< .001	(E1 = E3) < E2
	3	−3.53	.001	–	30.80	< .001	E1 > (E2 = E3)
	4	−7.71	< .001	–	7.13	.001	E1 < E2
	5	−5.43	< .001	–	6.82	.002	(E1 = E3) > E2
	**1**	**2.85**	**.005**	**+**	**5.90**	**.004**	**(E1 = E2) < E3**
	**2**	**−7.87**	**< .001**	**–**	**14.10**	**< .001**	**(E1 = E3) < E2**
	**3**	**−3.54**	**.001**	**–**	**30.82**	**< .001**	**E1 > (E2 = E3)**
	**4**	**−7.73**	**< .001**	**–**	**7.09**	**.001**	**E1 < E2**
	**6**	**−6.89**	**< .001**	**–**	**6.91**	**.001**	**(E1 = E3) > E2**
	1	2.82	.005	+	5.66	.004	(E1 = E2) < E3
	2	−7.88	< .001	–	14.17	< .001	(E1 = E3) < E2
	3	−3.56	.001	–	30.86	< .001	E1 > (E2 = E3)
	4	−7.76	< .001	–	7.05	.001	E1 < E2
	5	−5.44	< .001	–	6.75	.002	(E1 = E3) > E2
	6	−5.89	< .001	–	2.29	.105	*n.s*.
5	1	2.75	.007	+	5.84	.004	(E1 = E2) < E3
	2	−7.92	< .001	–	14.10	< .001	(E1 = E3) < E2
	3	−3.59	< .001	–	30.85	< .001	E1 > (E2 = E3)
	4	−7.82	< .001	–	7.08	.001	E1 < E2
	6	−6.95	< .001	–	6.90	.002	(E1 = E3) > E2
	7	−4.88	< .001	–	2.80	.064	*n.s*.
	**1**	**2.81**	**.006**	**+**	**5.43**	**.005**	**(E1 = E2) < E3**
	**2**	**−7.88**	**< .001**	**–**	**14.18**	**< .001**	**(E1 = E3) < E2**
	**3**	**−3.56**	**.001**	**–**	**30.47**	**< .001**	**E1 > (E2 = E3)**
	**4**	**−7.73**	**< .001**	**–**	**6.87**	**.001**	**E1 < E2**
	**6**	**−6.92**	**< .001**	**–**	**6.75**	**.002**	**(E1 = E3) > E2**
	**8**	**1.19**	**.237**	***n.s*.**	**7.66**	**.001**	**E1 > (E2 = E3)**
	1	2.80	.006	+	5.35	.006	(E1 = E2) < E3
	2	−7.88	< .001	–	14.18	< .001	(E1 = E3) < E2
	3	−3.56	.001	–	30.50	< .001	E1 > (E2 = E3)
	4	−7.73	< .001	–	6.86	.001	E1 < E2
	6	−6.92	< .001	–	6.73	.002	(E1 = E3) > E2
	7	5.69	< .001	–	2.65	.074	*n.s*.
	8	0.48	.634	*n.s*.	4.92	.009	E1 > E2

*Note*. Multiple comparisons: E1: auditory white noise experiment; E2: auditory oddball experiment; E3: IAPS pictures. The best model of each step is marked in bold. RF = response function; *t* test = test for the general direction of the response across experiments; direction of response = direction of the general response across all experiments (minus signs indicate accelerations, plus signs indicate decelerations); ANOVA = analysis of variance testing for a main effect of experimental condition.

### 3 Model Constants

Model constants are depicted in Table 5.

**Table 5:** Model constants

Response function	Parameters of Gaussian function	
	μ	σ
1	1	1.9
2	5.2	1.9
3	7.2	1.5
4	7.2	4
5	12.6	2
6	18.85	1.8

*Note*. μ = mean; σ = standard deviation.
